# Silicone Resin Applications for Ceramic Precursors and Composites

**DOI:** 10.3390/ma3063518

**Published:** 2010-06-02

**Authors:** Masaki Narisawa

**Affiliations:** Graduate School of Engineering, Osaka Prefecture University / 1-1, Gakuen-Cho, Naka-Ku, Sakai 599-8531, Japan; E-Mail: nar@mtr.osakafu-u.ac.jp; Tel.: +81-72-254-9312; Fax: +81-72-254-9912

**Keywords:** silicones, ceramic precursors, silicon oxycarbides, silicon chemistry, high temperature reaction, composites

## Abstract

This article reviews the applications of silicone resins as ceramic precursors. The historical background of silicone synthesis chemistry is introduced to explain the production costs and supply availability of various silicones. Thermal degradation processes of silicones are classified in terms of the main chain structure and cyclic oligomer expulsion process, which determine the resulting ceramic yield and the chemical composition. The high temperature decomposition of Si-O-C beyond 1,400 °C in an inert atmosphere and formation of a protective silica layer on material surfaces beyond 1,200 °C in an oxidative atmosphere are discussed from the viewpoints of the wide chemical composition of the Si-O-C materials. Applications of the resins for binding agents, as starting materials for porous ceramics, matrix sources with impregnation, fiber spinning and ceramic adhesions are introduced. The recent development of the process of filler or cross-linking agent additions to resin compounds is also introduced. Such resin compounds are useful for obtaining thick coatings, MEMS parts and bulk ceramics, which are difficult to obtain by pyrolysis of simple organometallic precursors without additives.

## 1. Introduction

The polymer precursor method has been mainly developed in the field of inorganic fibers. Carbon fibers with high strength were developed in the early years, and silicon carbide fibers were invented in 1970s [1,2]. The high heat resistance of SiC fibers, even in an oxidative atmosphere, promoted the synthesis chemistry of various ceramic precursors, like polycarbosilanes, polysilazanes and the recently successful polyborosilazanes [3,4]. On the other hand, the high temperature resistance of ceramic fibers derived from polycarbosilanes is being continuously improved, even at present [5,6,7,8]. Such advanced SiC based fibers are mainly used as reinforcements in ceramic matrix composites with extremely high heat resistance.

On the other hand, sol-gel methods for oxide base ceramic synthesis have been developed over the past few decades. Various alkoxides are now commercially available, and papers on the theme of sol-gel science and technology are being published at a rate of at least 4,000 per year.

When we contemplate such a situation, it is evident that the combination of carbide base ceramics and oxide base ceramics by some chemical technique will be a great issue, which will be final target of the organic–inorganic hybridization process. We, however, remember simultaneously that such hybridization processes have already been accomplished with great success many years ago. Just around the time of World War II, various silicone polymers, the polysiloxanes, were synthesized on a large scale and widely commercialized. They are available as electric insulator coatings, surface treatments for glass materials, heat resistant oils and chemically stable elastomers. Now such products are highly sophisticated industrial commodities, and we often forget the chemical background of the various commercialized silicone resins. It is a shame that the role of silicones in ceramic technology is reduced to that of being somewhat muddy additives for shaping the starting materials before the sintering process. Here, I tried to shed light again on the classic silicone polymer science, which is quite fundamental in organometallic chemistry and high temperature organic - inorganic reactions. Recent activity in the field and renewed interests in Si-O-C materials correctly indicate the great importance of the modern silicones as ceramic precursors.

## 2. Historical Background of Silicone Resin Production [9,10]

Silicon is industrially produced at present on a tremendous scale. The origin of silicon is high purity mineral silica sand. Such silica sand is typically reduced by carbon in an electric arc furnace at 3,000 °C. The silicon obtained, of 98–99% purity, is called “metal-grade silicon”. Besides the uses in the electronics and silicone polymer industry, such metal-grade silicon is used as an important component in various alloys of Fe, Al or Mg.

For the uses in electronics, the metal grade silicon is reacted with SiCl_4_ and hydrogen to yield HSiCl_3_. This compound is a transparent liquid with a boiling point of 31.8 °C. The liquid nature of HSiCl_3_ means that the compound can be distilled for purification. After the distillation, reduction by hydrogen and the Czochralski process, a single crystal of silicon with extreme high purity is obtained. The unique properties of the pure silicon, such as its semi conductive nature, acceptance of doping and formation of insulating silica layers on the surface during oxidation, are presently well known. The application of Si for solar cells is increasing its importance in recent times.

SiCl_4_ is an important recycled product in the pure silicon industry. SiCl_4_ is also useful for the synthesis of various organosilicon compounds. Kipping systematically investigated organosilicon monomers and polymers derived from SiCl_4_. The first step is the reaction of a SiCl_4_–Grignard reagent (RMgBr). After the reaction, Si-Cl bonds are substituted by Si-R bonds. In the case of C_2_H_5_MgBr, the chemical reaction is expressed by a following equation;

SiCl_4_ + C_2_H_5_MgBr → Si(C_2_H_5_)Cl_3_ + MgBrCl (1)

Of course, the Grignard reagent attacks not only the Si-Cl bonds in SiCl_4_, but also the Si-Cl in Si(C_2_H_5_)Cl_3_ and Si(C_2_H_5_)_2_Cl_2_. Therefore, the products derived from SiCl_4_-C_2_H_5_MgBr combinations are often mixtures of various Si(C_2_H_5_)_x_Cl_4-x_ species. Each compound must be isolated from the mixture by fractional distillation.

Kipping also found that the isolated organosilicon compounds with Si-Cl bonds easily reacted with water. In the case of R_2_SiCl_2_ including Si(C_2_H_5_)_2_Cl_2_, the reaction is expressed by a following equation:

SiR_2_Cl_2_ +2H_2_O → SiR_2_(OH)_2_ + 2HCl (2)

When R is not bulky, the silanol groups are simultaneously condensed, and a polysiloxane with a Si-O backbone structure is obtained:

nSiR_2_(OH)_2_ → (-SiR_2_-O-)_n_ + nHCl (3)

Some part of the silanols remains as terminal groups, and some oligosiloxanes with ring structures can also be obtained in the resulting mixtures. The first silicone resin, reported by Kipping as a “glue-like” product, did not however attract any kind of industrial attention in those days.

On the other hand, the polymeric nature of these “glue-like” silicones attracted the attention of Hyde and related groups at Corning Glass Works. The company developed the industrial process for silicone resin production on the basis of the SiCl_4_—Grignard reagent combination, and opened the doors to silicone resin commercialization. The silicone resins were found to be highly compatible with glass materials. Utilization as binders for glass fibers and scratch resistant coatings on glass plates was promoted. The main silicone investigated by Hyde was a kind of polyethylphenylsiloxane (PEPhS).

The synthesis of PEPhS with using Grignard reagent is, however, a multi-step process. Grignard reagents are highly flammable, and the synthesis requires a large amount of metallic magnesium. The silicone thus obtained is rather special and a little far from conventional plastic, like polyethylene, polyamide or phenolic resins, widely produced from petroleum industry raw materials.

In order to overcome such economical and industrial problems, Rochow developed at General Electric a direct synthesis process for organosilicon monomers without the aid of Mg in 1940. This was essential progress in the silicone industry.

He got idea from a following reaction of SiHCl_3_ formation from Si and HCl:

Si (s) + 3HCl (g) → SiHCl_3_ (g) + H_2_ (g) (4)

In the Rochow process, CH_3_Cl was introduced in a reaction column instead of HCl, and grains of Cu-Si alloy were loaded in the column. Cu was expected to act as a catalyst. A liquid product, a mixture of methylchlorosilanes, is obtained by the following reaction:

Excess CH_3_Cl (g) + Si (s) → Si(CH_3_)_2_Cl_2_ (L) + Si(CH_3_)Cl_3_ (L) + SiHCl_3_ (g, L) + SiH(CH_3_)Cl_2_ (L) + Si(CH_3_)_3_Cl (L) + SiCl_4_ (L) (5)

This is intrinsically a gas-solid reaction. Thus, continuous operation is possible by adjusting the rate of introduction of CH_3_Cl gas, Si, and Cu powders into a reactor. By fractional distillation, each compound can be isolated with high purity. Si(CH_3_)_2_Cl_2_ has the highest boiling point, while Si(CH_3_)_4_ has the lowest boiling point, except for the starting CH_3_Cl (Table 1). Si(CH_3_)_2_Cl_2_ is the most valuable component in the obtained mixture, because it forms linear Si-O-Si chains after the hydrolysis. Any kind of methylchrolosilanes are, however, useful for tailoring silicone resins, oils, greases, rubbers and varnishes.

**Table 1:** Boiling points of methylchlorosilanes found in the Rochow process product.

Compound	Boiling Point (°C)
CH_3_Cl	−24.2
(CH_3_)_4_Si	27.5
HSiCl_3_	31.8
(CH_3_)HSiCl_2_	40.7
(CH_3_)_3_SiCl	57.3
SiCl_4_	57.6
(CH_3_)SiCl_3_	65.7
(CH_3_)_2_SiCl_2_	70

On the other hand, phenyl chlorosilanes are also useful monomers in the silicone industry. It is possible to synthesize phenyl chlorosilanes by direct reaction of chlorobenzene (C_6_H_5_Cl) and the Si-Cu alloy. Higher temperature (400–500 °C) and a larger amount of Cu content (30 mass %) are however required.

Compared to such direct syntheses using the Si-Cu alloy, the following dehydration or dehydrochloration reaction is more efficient and widely available for the production of phenyl chlorosilanes and vinyl chlorosilanes:

C_6_H_6_ + HSiCl_3_ → C_6_H_5_SiCl_3_ + H_2_ (6a)

CH_2_=CHCl + HSiCl_3_ → CH_2_=CHSiCl_3_ + HCl (6b)

The introduced HSiCl_3_ is obtained by the fractional distillation of the product of Rochow process, or is obtained from the products in the pure silicon industry. From a cursory glance at such chemical processes, we can get a sense of how polymers so unique as the silicones have been widely produced at relatively low cost, and what kind of silicone is more popular from the viewpoint of the industry.

## 3. Thermal Degradation of Linear Silicones

Silicone is superior in heat and chemical resistance as compared with ordinary polymers. Silicone oils are necessary component in high vacuum systems, and we often see elastic silicone rubber materials in medical and chemical uses. In most cases, linear polysiloxane or partly cross-linked polysiloxane were used in commercialized products. Si-O bonds in siloxane chain are flexible as compared with C-C bonds, and silicones intrinsically maintain their liquid nature over a wide temperature region. For example, a glass transition temperatures of polydimethylsiloxane (PDMS) or polymethylphenylsiloxane (PMPhS) are −127 °C and –86 °C, respectively [11,12].

**Figure 1:**
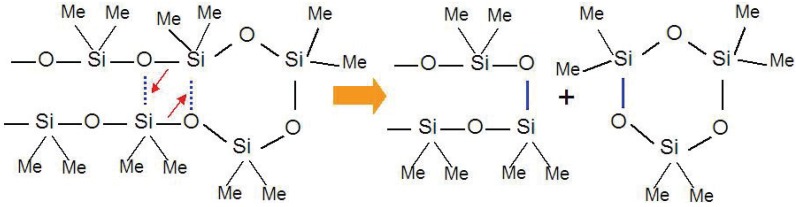
Cyclic oligomer (trimer) expulsion mechanism from a polymethylsiloxane chain during thermal decomposition process.

Thermal degradation of PDMS with complete linear structure proceeds at 290–600 °C with formation of cyclic oligomers. In an inert atmosphere or vacuum, a trimer (Si_3_O_3_(CH_3_)_6_) and tetramer (Si_4_O_4_(CH_3_)_8_) are the major components in the decomposition gas. Chemical species with higher molecular weight, like a hexamer and an octamer, are also found as components. Thomas *et al*. proposed an intramolecular cyclization process for the thermal degradation of PDMS [13]. The low activation energy, 40 kcal/mol, suggests the existence of a stable transition state, which promotes the degradation of the silicone resin. The linear siloxane chains can easily make intramolecular contact because of the flexible nature of the chains and the Si d-orbital interactions. Thus, the degradation proceeds by simultaneous rearrangement of Si-O bonds with expulsion of cyclic oligomers (Figure 1).

It is interesting that PDMS is obtained not only from hydrolysis of (CH_3_)_2_SiCl_2_ but also from ring opening polymerization of the cyclic tetramer (Si_4_O_4_(CH_3_)_8_). In other words, the decomposition process of PDMS is a kind of depolymerization.

In the presence of oxygen, the degradation process of linear silicones becomes complex [14]. In a relatively low temperature region, oxygen acts as a catalyst promoting the scission and rearrangement processes of the Si-O bonds. Thus, the depolymerization process and removal of volatile oligomers occurs at a relatively low temperature (290 °C) as compared with that in an inert atmosphere (400 °C). The residual siloxane is, however, condensed by oxidative cross-linking, which reduces the mass loss rate in the last stage of the decomposition. Such oxidation cross-linking is possibly triggered by the formation of radicals on side groups, which trap oxygen and form peroxides, which sometimes accelerate the cross-linking and sometimes accelerate the depolymerization process. In an oxidative atmosphere, ca. 10 mass % of silica is obtained as a result of the competition between oxidative cross-linking and volatilization of oligomers.

Even in an inert atmosphere, an inorganic residue is sometimes obtained after pyrolysis with very high heating rates [14]. Perhaps, condensation of low molecular weight oligomers, which cannot be diffused out, takes place. The obtained glassy black product is thought to possess Si-O-C composition.

The decomposition processes of various linear polymers composed of -SiR_1_R_2_-O-, -Si(CH_3_)_2_-CH_2_-Si(CH_3_)_2_-O- or -Si(CH_3_)_2_-CH_2_-CH_2_-Si(CH_3_)_2_-O- units were also investigated by Thomas *et al*. [15]. Thermal degradation proceeds *via* the depolymerization reaction and the mechanism is similar to that of the usual linear polysiloxanes like PDMS. The major gaseous products are cyclic trimers and tetramers.

Introduction of phenyl groups in silicone polymers usually increases the onset temperature of mass loss of polysiloxanes [15,16,17]. The residual mass at 1,000 °C, however, does not increase, because of evolution of benzene, toluene and cyclic siloxane oligomers during higher temperature degradation processes at 400–600 °C. Siloxane oligomers with phenyl groups are absent in the gaseous products. Perhaps, phenyl side groups are decomposed by the radical reaction, and the cyclic oligomer expulsion follows such side group decomposition processes. When the chain is flexible, anyway, it is likely difficult to avoid the cyclic oligomer expulsion caused by the Si-O and Si-C bond rearrangements during the thermolysis.

## 4. Increased Ceramic Yield in Cross-Linked Silicones

In order to increase the ceramic yield of silicones, a dense cross-linked structure, which prevents the bond rearrangement process during heating, is necessary. As compared with the C-O (351.5 kJ mol^−1^), C-C bond (347.7 kJ mol^−1^) and C-Si bonds (290.0 kJ mol^−1^), the high energy of the Si-O bond (369.0 kJ mol^−1^) is promising for increasing the ceramic yield of silicones. Introduction of vinyl, phenyl groups or acetylene linkages in silicone molecular structure may be also effective for increasing the apparent ceramic yields. The efficiency of such side groups, however, often depends on behavior of “radicals”, which sometimes decompose side groups to gaseous product. There is also the concern that during pyrolysis such side groups are converted to free carbon domains, which are not directly incorporated into the inorganic Si-O-C networks.

Zhou *et al*. introduced T units in siloxane chains in order to investigate the effect of Si-O cross-linking on the thermal stability [17]. As the content of T units increases, the resulting ceramic yield increases. On the other hand, Mantz *et al*. investigated thermolysis of polyhedral oligometric silsesquioxane (POSS)–siloxane copolymers [18]. Loss of the cyclic dimethylsiloxane oligomers proceeds at 400 °C, and loss of the silsesquioxane “cage” structure proceeds at 450–650 °C. This means that the incorporated cage structure, composed of complete T units, is not simply maintained during the heating, and the thermal degradation process in this case is complex, which possibly corresponds to some steric hindrance effect of the cage structure on the main chain rearrangement process.

Burns *et al*. (Dow Corning) synthesized various cross-linked polysiloxanes by sol-gel methods, and investigated their ceramization process [19]. The starting monomers are PhSi(OMe)_3_, MeSi(OMe)_3_ and (ViMe_2_Si)_2_O. After the condensation reactions of the monomers in controlled amounts, the residual OH groups are terminated by ViMe_2_SiCl. An expected application of these silicones is as a sintering aid in the SiC base ceramics production process. The silicones are also expected to help the molding, pressing, casting and infiltration processes of prepared slurries composed of SiC grains, silicones and a small amount of solvent. Ceramic yields of the obtained polymers at 1,100 °C in an inert atmosphere are ca. 65–75 mass%, while O/Si ratios are 1.3–1.4. Carbon contents in these silicones are always controlled at high values. The silicones are converted to amorphous SiO_x_C_y_ by pyrolysis at 1,100 °C, and successively converted to SiC and excess carbon by carbothermic reduction at 1,800 °C expressed by the following reaction:

SiO_x_C_y_ → SiC + xCO + (y-x-1)C (7)

Residual masses after the carbothermic reduction process are 35–50 mass %. The yield tends to increase with the increase in the carbon content in the starting Si-O-C. The amount of excess carbon after pyrolysis at 1,800 °C also increases. SiC nanocrystallites formed *in situ* and excess carbon are expected to act as sintering aids for the loaded coarse SiC grains during high temperature heat treatment.

Hurwitz *et al*. carried out systematic studies on the pyrolysis process of polyphenyl–polymethyl–silsesquioxane co-polymers, which were synthesized by hydrolytic condensations of PhSi(OMe)_3_ and MeSi(OMe)_3_ [20]. The ceramic yields of the co-polymers decreases as the phenyl content in the co-polymer increases, while the ceramic yield of polyphenylsilsesquioxne (PPSQ) is ca. 60%, and that of poymethylsilsesquioxane (PMSQ) is ca. 80%. The onset temperatures of mass losses of PPSQ and the co-polymers is 500 °C, while that of PMSQ, with 100% methyl side groups, is 750 °C.

Oxidation resistance of Si-O-C materials derived from co-polymers of 30P, 50P and 70P (P means a phenyl molar content to phenyl + methyl groups in the co-polymer) were also investigated [20]. Carbon burning usually proceeds in the temperature range of 600–1,000 °C. As the phenyl group content decreases, however, the oxidation resistance of the Si-O-C materials increases, which is indicated by slow mass loss rate in a TG curve.

Brewer *et al*. (Dow Corning) investigated the oxidation resistance of Si-O-C materials derived from PhSi(OMe)_3_, MeSi(OMe)_3_ and (ViMe_2_Si)_2_O [21]. The oxidation resistance possesses a close relationship to the carbon content in the Si-O-C materials. The Si-O-C materials derived from the co-polymer of 0.78 MeSi(OMe)_3_ and 0.22 (ViMe_2_Si)_2_O (with a chemical composition of SiO_1.35_C_1.14_) shows the highest oxidation resistance. Even after 500 h treatment in air at 1,200 °C, the residual carbon content exceeds half the original content. It is regrettable that the observed oxidation resistance is not based on complete quantitative estimation, because the specific surface area of prepared powder-like sample is not defined, although Brewer observed formation of a transparent oxide layer on the Si-O-C materials after the oxidation treatment, which should protect inner materials from rapid oxidation.

The O/Si molar ratios in the densely cross-linked silicones, described in this section, are expected to be 1.0–1.5 before pyrolysis. Wilson *et al*. (Dow Corning) investigated the chemical compositions of a series of Si-O-C materials derived from cross-linked silicones [22]. The starting O/Si ratios are roughly maintained when the ceramic yields of the resins are high. In some cases with relatively low ceramic yields, the O/Si ratios are increased during pyrolysis. Such an increase in O/Si ratio can be simply explained by removal of a large amount of siloxane oligomer during pyrolysis, because the O/Si of the cyclic siloxane oligomers is 1.0. The decrease in the O/Si ratio during pyrolysis observed in a few cases is difficult to explain. The evolution of the “cage” structure (O/Si molar ratio of 1.5) is one mechanism to explain such decreases, but there is no sufficient data indicating the “cage” sublimation during the pyrolysis of the cross-linked silicones. Quite recently, Ionescu *et al*. reported evolution of octamethyl T8 polyhedral oligosilsesquioxane (POSS) during cross-linked silicone resin pyrolysis [23]. Quantitative estimation of the evolved “cage” amount and influence on total chemical composition of ceramic reside are, however, still ambiguous at present.

In an inert atmosphere, once formed SiO_x_C_y_ materials from the silicone resins are considered to be decomposed beyond 1,400 °C. This expectation is based on the carbothermal reduction process of silica-carbon mixtures. In other words, chemical activity of oxygen in the SiO_x_C_y_ is assumed to be almost equal to that in silica. If the carbon content in the Si-O-C material is sufficiently high or pyrolysis environment is rather closed, SiC and excess carbon are formed during the carbothermnal reduction, as shown in Equation (7).

If the carbon content in the Si-O-C material is low or evolved gas from the system easily diffuses out, the gasification of the Si-O-C material shown in the following equation would become dominant:

SiO_x_C_y_ → SiO + (x − 1)CO + (y − x + 1)C (8)

The balance between equations (7) and (8) depends not only on the starting carbon content but also on the partial pressures of CO and SiO in the surrounding environments. In addition, vapor phase condensation reactions of SiO, CO and C are known to form various kinds of SiC-SiO_2_ whiskers or nano tubes. Thus the whole chemical process depends on the partial pressure of the individual gaseous species present and often becomes quite complex.

On the other hand, the SiO_x_C_y_ material is expected to be converted to silica when the partial pressure of oxygen in the heat treatment condition is sufficiently high:

SiO_x_C_y_ + (1 − x + 2y)O_2_ → SiO_2_ + yCO_2_ (9)

**Figure 2:**
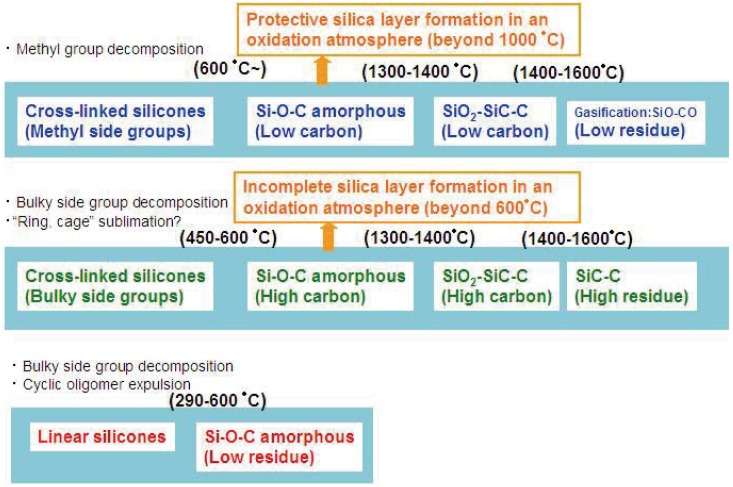
Various modes of thermal decomposition of silicone resins.

Such silica is often formed as a layer on the material surface. The completeness and the efficiency of the formed layer for the material protection also depend on the carbon content in the materials and oxygen activity in the surrounding environment. Figure 2 summarizes the various thermal decomposition modes of silicones or silicone-derived amorphous species. In the low temperature region, the starting chain structure determines the degradation process. In particular, “linear” or “branched” is important. In the high temperature region, the chemical composition and the pyrolysis atmosphere are the major factors which determine the degradation mode.

## 5. Industrially Available Silicone Resins with High Ceramic Yields

As shown in a previous section, polymethylsilsesquioxane (PMSQ) is a simple and promising ceramic precursor for Si-O-C base materials because of the low carbon content and intrinsic low cost. In early days, however, PMSQ obtained by condensation reactions was not soluble and stable. In 1978, the first soluble PMSQ in resin form was synthesized by Suminoe [24,25]. In his process, (C_2_H_5_)_3_N and MeSiCl_3_ are dissolved in mixed solvent of methylisobutylketone (MIBK) and tetrahydrofuran (THF). The hydrolysis reaction is carried out by adding water dropwise to the solution. After temporary precipitation of a white salt and re-dissolution of the salt by increasing the amount of water, the obtained solution is heat-treated and refluxed at 100–110 °C. After extraction, washing and re-precipitation, a resin with a molecular weight of 9,000 is obtained. It is stable even after one month storage, and soluble in organic solvents like THF or toluene. It is also possible to obtain PMSQ with higher molecular weights. The low molecular weight PMSQ is dissolved in ether with water and Me_3_NHCl, and hydrolyzed again by heating at 130 °C for 4 h. The measured molecular weight of the resin is increased to 100,000. The solubility is organic solvents is also maintained. Such solubility possibly depends on an amount of the residual OH groups in the structure, which do not completely disappear after the hydrolysis reactions.

Nowadays, a few kinds of silicone resins are commercialized under the PMSQ name. Wacker-Belsil PMS MK^TM^ is available from Wacker Chemie AG. It is white powder with the proposed chemical structure (-(CH_3_)Si(O_3/2_)-)_n_ [26]. The softening point is 50–60 °C and the resin accepts the attack of alcohol. Since the PMSQ reported by Suminoe is washable with methanol, the molecular weight of PMS MK^TM^ is possibly low and it contains a large amount of OH groups as compared with the one previously reported.

YR 3370^TM^ is available from Momentive Performance Materials Japan. It is a transparent hard resin that softens at ca. 110 °C. Since the YR3370^TM^ is also soluble in ethanol, the structure also probably contains a considerable amount of OH groups. The hard appearance, however, suggests a higher molecular weight of YR3370^TM^ than that of PMS MK^TM^ powder. The elemental analysis of YR3370^TM^ shows the chemical composition of SiO_1.78_C_1.22_H_3.67_. The content of carbon is a little high, beyond the expectation based on an assumed [-(CH_3_)Si(O_1.5_)-] unit structure.

**Figure 3:**
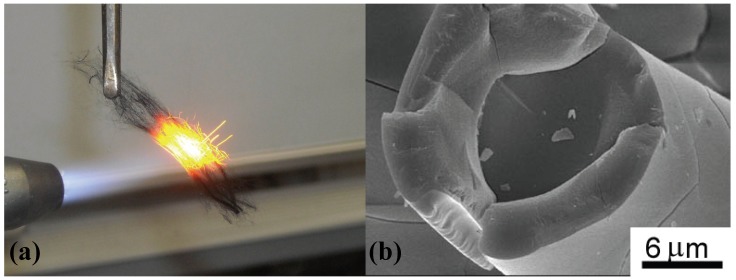
(a) Screening test on heat resistant ceramic fibers (result of the Si-O-C (SiO_1.5_C_0.63_) fiber, 20 min) [29]; (b) residual Si-O-C core surrounded by a thick silica layer after continuous 24 h oxidation at 1,511 K on the Si-O-C fiber.

Kim *et al*. used polymer blend or filler loaded YR 3370^TM^ as starting materials for SiC or silicon oxycarbide based porous ceramics [27,28]. Control of the starting porous structure depends on the viscoelastic nature of the resin, which was analyzed in detail by the same group [28]. By adjustment of the conditions, even the melt spinning process is available for YR 3370^TM^ at 130–180 °C. The resin fiber can be converted into oxidation resistant continuous Si-O-C ceramic fibers by adjusting the conditions of metal chloride vapor curing and the pyrolysis conditions [Figure 3 (a) and (b)] [29].

**Figure 4:**
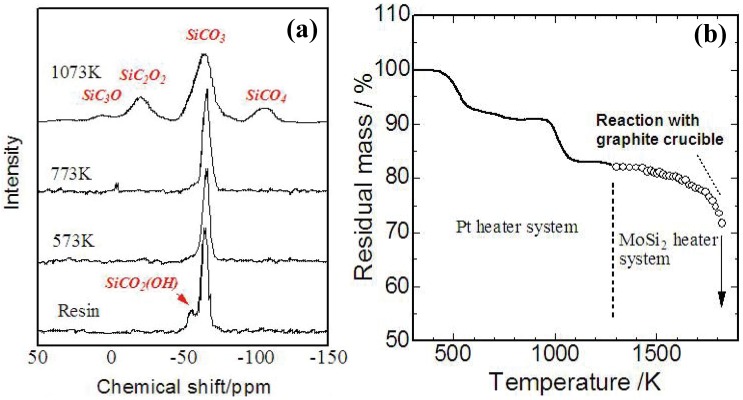
(a) Results of TG analysis on YR3370^TM^ resin in carbon rich atmosphere (10 K/min up to 1473 K, 3 K/min beyond 1473 K and holding at 1873 K for 3 h) [29]; (b) ^29^Si-NMR spectrum of YR3370^TM^ pyrolyzed at various temperatures [30].

The ceramization of YR3370^TM^ was also well characterized [29,30]. While the viscoelastic properties of the commercialized or synthesized PMSQs are various, the ceramization process of PMSQs is not sensitive to the synthesis conditions. Figure 4 (a),(b) shows a TG curve and ^29^Si-NMR spectrum of YR3370^TM^ during the pyrolysis. A 5% mass loss occurs at 250–300 °C with cross-linking of OH groups, and an 8% mass loss occurs at 600 °C with evolution of methane. At 1,200 °C, the analyzed chemical composition of the amorphous Si-O-C was SiO_1.5_C_0.68_. The mass loss observed beyond 1,400 °C corresponds to gasification of Si-O-C. Reaction between a graphite crucible and the Si-O-C amorphous possibly enhances the continuous mass loss during the holding at 1873 K. These behaviors in ceramization are consistent with the previous data reported about various PMSQs [20,31].

SR 350^TM^, which is a kind of silicone resin with low carbon content and with highly branched structure, is available from General Electric Silicone Products. The resin is synthesized from 2–8% of dimethyldichlorosilane, Si(CH_3_)_2_Cl_2_, and 92–98% of methyltrichlorosilane, Si(CH_3_)Cl_3_ [32,33]. The resin is softened at 30 °C and becomes fluid at 90 °C. SR350^TM^ is also soluble in most organic solvents like toluene, ketones, alcohols and ether. In the pyrolysis of SR350^TM^, a 15 mass %mass loss is observed up to 200 °C. Perhaps, the amount of the residual hydroxyl groups is high as compared with complete PMSQ resins, and the OH groups are condensed up to 200 °C with H_2_O evolution. The ceramic yield in an inert atmosphere is 75 mass % at 1,000 °C. Oxidation resistance of the Si-O-C materials derived from SR 350 ^TM^ is also reported in terms of protective silica layer formation on the material surfaces.

Some resins with the PMSQ name are available mainly in the field of cosmetics. Gransil PSQ^TM^ is supplied by Grant Industries, and Tospearl^TM^ ie supplied from Momentive Performance Materials Japan. The molecular structure of Tospearl^TM^ is possibly similar to that of YR3370^TM^, but the shape is often micro spherical and it is insoluble in any solvents. There is no reported ceramization data for these PMSQs.

## 6. Application of Silicone Resins for Various Ceramic Products

Hurwitz reported early studies about the utilization of polysilsesquioxanes (PSQ) in the field of ceramic matrix composites [34,35]. The viscoelastic properties of PSQ copolymers (with CH_3_-, C_3_H_7_- and C_6_H_5_- side groups) were adjusted to make the melt spinning process possible at 70–100 °C. The spun fiber accepted UV curing with the decomposition of phenyl side groups. The infiltration and pyrolysis of the co-polymer in SiC fiber (Nicalon) fabrics was also examined for making heat resistant CMC. In an inert atmosphere, heat resistance of the formed CMC up to 1,400 °C was suggested. The pyrolysis of such copolymers including phenyl groups, however, must accept the increased amount of residual carbon in the obtained Si-O-C materials. It is probably the weak point of the process because the high carbon content often diminishes the oxidation resistance of the Si-O-C amorphous obtained after pyrolysis.

The synthesis of heat resistant ceramic foams by using the viscoelastic nature of silicone resins was widely examined. Colombo *et al*. reported Si-O-C ceramic foam synthesis from the mixtures of SR350, monomers of polyurethane (polyols and diamines) and a blowing agent (CH_2_Cl_2_: b.p. of 40 °C) [36]. Since the silicone resin mass ratio to polyurethane (PU) was 1.0, SR350 was expected to be expanded as concentrated polymer solution and solidified with PU during the foaming process. The reaction of silanol groups in SR350 with amino groups in the diamine, however, was possible and the resulting foaming process may be more complex. Anyway, the silicone resin network displayed good compatibility with PU during the whole process, because the tailored foam structure was maintained after pyrolysis up to 1473 K. The Si-O-C ceramic foams with a bulk density of 0.1–0.4 gcm^−3^ and cell size of 300–600 mm were obtained. On the other hand, direct foaming process of preceramic polymers (PCS-YR3370) by using CO_2_ gas saturation—desaturation process was reported by Kim *et al.* [37]. CO_2_ has a critical point close to room temperature and is easily dissolved in polymer networks by loading under high pressure. By rapidly dropping the CO_2_ pressure, a number of fine cells (<10 μm) were formed in the resin matrix. After the pyrolysis, the Si-O-C microcellular structure was obtained. Another way to achieve such a Si-O-C microcellular structure was the use of sacrificial fillers of PMMA microbeads [38]. In this method, control of long range pore order is possible in principle by packing of microbeads. On the other hand, Zeschky *et al*. reported the control of porosity gradient in precursor foam by adjusting the viscosity of the melted precursor media [39]. A major component of the foamed media was a kind of methyl-phenyl-silsesquioxane (Sirless H44, Wacker Chemie). The media, however, contains considerable amounts of Si and SiC particles dispersed in the resin melt. Therefore, the whole thermoplastic–thermosetting nature during the heating and holding was complex. Bubbles of H_2_O-C_2_H_5_OH derived from condensation reactions of the resin terminal groups were desaturated, coarsened and rose up to surface during the heating. Such a structure solidified in the middle of the bubble rising process. A ceramic body with hierarchical, nano-meso-porosity was synthesized by Colombo *et al.* [40]. In the reported process, mesoporous silica was deposited on the Si-O-C microporous ceramics derived from Wacker-Belsil PMS MK^TM^ and PMMA microbeads. Deposition of mesoporous silica coating on the Si-O-C wall was carried out by infiltration and pyrolysis of solution of tetraethyl orthosilicate (TEOS) and block copolymer. The resulting specific surface area was one order of magnitude higher than that in previous Si-O-C porous ceramics.

Silicone resins are thus able to accept various kinds of fillers, plasticizers, blowing agents and cross-linking agents. Control of cell size, density and connectivity of foamed cells are intrinsically possible by using various methods developed in the plastic foaming industry field. The reported excellent dimensional stability after the pyrolysis is possibly based on the thermosetting nature and high ceramic yield of the silicones with dense Si-O cross-links.

On the other hand, heat resistance of the resulting Si-O-C porous bodies is a difficult problem to be summarized simply. If additional organic agents were completely burned out during the pyrolysis, the resulting heat resistance would be high. Rouxel *et al*. evaluated high temperature viscosity of Si-O-C amorphous materials from bending creep displacement of thin small rods with no significant defects. The estimated viscosity values were two orders of magnitude higher than that of pure silica [41]. The carbon content in the Si-O-C amorphous was higher, and the resulting viscosity was higher. Incorporation of carbon in silica-like network is thought to be key factor, which inhibits the viscous flow of the silica-like networks [41,42,43]. Besides the carbon network, progress of the crystallization of β-SiC and β-SiO_2_ in the Si-O-C matrix harden the materials significantly. In addition, the formation of a protective silica layer on the Si-O-C surface is expected to prevent rapid oxidation.

If the organic additives were to remain in the pyrolyzed materials, however, carbon domains derived from the additives may cause carbon rich domains in the pyrolyzed Si-O-C. Carbon rich domains often diminish the oxidation resistance by breaking the protective silica layer, or diminish the high temperature strength in an inner atmosphere by promoting the evolution of CO and SiO. If the starting precursor contains SiC grains, however, the reaction between Si-O-C and carbon domains may promote a sintering–binding process of the SiC fillers during the pyrolysis process. Perhaps, the upper limit of heat or environmental resistance of the pyrolyzed materials varies depending on the intrinsic chemical compositions and resulting microstructures.

**Figure 5:**
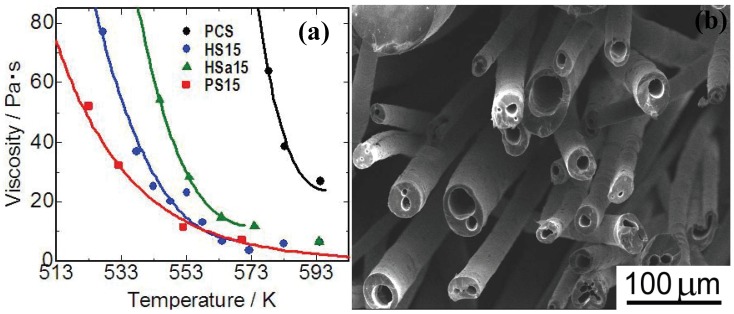
(a) Viscosity—temperature relationship of the polymer blends; PCS: polycarbosilane, HS15: PMHS 15 mass %to PCS, Hsa15: PMHSa (PMHS with low Si-H content) 15 mass %to PCS, PS15: PMPhS 15 mass %to PCS; (b) Silicon carbide base micro tubes derived from melt - spun polymer blend of polycarbosilane–polymethylhydro siloxane) (from the Ph.D. thesis of K. Kita, Osaka Prefecture University, 2010).

Blends of silicones with ordinary ceramic precursors, like polycarbosilanes, polysilazanes and polysilanes, are another interesting theme. For example, polycarbosilane (PCS), which is famous as a precursor of continuous silicon carbide base fibers, accepts the dissolution of various silicone oils [44,45]. Since main chain structure of silicones (Si-O-Si) is different from the chain structure of PCS (Si-CH_2_-Si), compatibility of the silicone oils to PCS is intrinsically limited. Up to 10 mass%, however, the oils act as plasticizers of the prepared blend polymer melts [Figure 5(a)]. Polymethylphenylsiloxane (PMPhS) addition shows the most remarkable plasticizer effect, which diminishes the macromolecular interactions between the polymer chains. On the other hand, the plasticizer effect of polymethylhydrosilixoane (PMHS) is limited because of decomposition of Si-H group during the heating. Continuous micro tubes, however, can be spun from the polymer melt by adjusting the H_2_ gas evolution, dissolution and desaturation processes in the melts. The synthesis of such micro tube is positioned as a unique and extreme case of the precursor polymer foaming process reviewed in previous sections. The spun micro tubes can be converted to SiC base micro tubes by the pyrolysis at 1273 K [Figure 5 (b)]. The process deeply depends on Si-H content in PMHS. Since the major precursor used in this system is PCS, high heat resistance is expected on the pyrolyzed micro tubes. 

As a quite recent topic, piezoresistive effect in Si-O-C ceramics derived from PMS MK^TM^ after 1,400 °C pyrolysis is reported by Riedel *et al*. [46]. The high strain sensitivity suggests the change of percolation network of excess carbon during the strain application. Perhaps, silicone resin application for ceramic precursor is not limited in the field of structural ceramics. The unique electrical properties of the pyrolyzed Si-O-C materials will be applied for functional ceramics.

## 7. Modification of Silicone Resins for Advanced Ceramic Precursors

The advanced science and technology in modern synthesis chemistry contribute to the production of various promising ceramic precursors with tailored compositions and unique molecular structures. In the plastics industry, however, it is notable that the various kinds of useful materials are produced from limited kinds of conventional polymers. Selection of additive, filler, blend technique and control of the cross-linking process are particularly important to produce materials with excellent properties at reduced cost. In spite of the absolute importance, systematic study of such basic techniques on using silicone resin as ceramic precursor is still at a quite early stage of progress.

Greil proposed the notion of “active fillers” in the field of the ceramic precursors. The basic idea is trapping of carbon and nitrogen from the surrounding matrix or the gaseous atmosphere by dispersed fillers [47]. The volume of the fillers increases during such carbonization–nitrogenation reactions, which compensates for the volume shrinkage of the surrounding matrix. In the reported successful cases, CrSi_2_ or MoSi_2_ powders are dispersed in a kind of poly(silsesquioxane) having C_6_H_5_-, CH_2_=CH‑, CH_3_- and H- side groups, and the mixed materials are pyrolyzed up to 1,500 °C in an nitrogen gas flow.

SiOC_x_ (High carbon amorphous) + CrSi_2_ + 2N_2_ (in the atmosphere) → 2/3Cr_3_C_2_ + Si_3_N_4_ + SiOC_y_ (Low carbon amorphous) (10a)

SiOC_x_ (High carbon amorphous) + MoSi_2_ + 2N_2_ (in the atmosphere) → 1/2Mo_2_C + Si_3_N_4_ + SiOC_y_ (Low carbon amorphous) (10b)

During the pyrolysis, silicon in the fillers is converted to Si_3_N_4_, while chromium and molybdenum in the fillers are converted to Cr_3_C_2_ and Mo_2_C, respectively. The carbon content in the starting matrix polymer is not defined precisely, but the absolute amount is sufficient to carbonize chromium and molybdenum by the evolved hydrocarbon gaseous compounds during the pyrolysis. This method is available in principal not only for silicones, but also for carbosilane (Si-C) or silazane (Si-N) backbone precursors.

On the other hand, it is easily predictable that trapping of oxygen from the surrounding silicone resin matrix is probably possible, if the reducing power of the selected filler is sufficiently high. In spite of the simplicity, however, there are not many practical examples based on such ideas. Colombo *et al*. reported the joining of SiC/SiC composites by the combination of SR350 and 88Al-12Si metal powder [48]. The importance of the Al-Si melting for increasing the joint strength and formation of Al_2_O_3_ and SiC at low temperature region are suggested. The observed shear strength exceeds that of merely pyrolyzed SR350 joined materials and the highest shear strength is observed at 1,200 °C heat treatment. The concrete characterization of the formed phases during pyrolysis, however, is absent in the written article.

Recently, our group investigated the ceramization process of the YR3370–metal Al particle composites [49]. When the YR3370 resin is simply pyrolyzed in an inert atmosphere, the resin is converted to SiO_1.5_C_0.68_ amorphous up to 1,000 °C. XRD patterns of the pyrolysis products suggest that the SiC formation proceeds at 1,400 °C with no significant mass loss. In theory the thermodynamically stable phase derived from SiO_1.5_C_0.68_ is a mixture of SiO_2_, SiC and free carbon:

SiO_1.5_C_0.68_ → 0.75SiO_2_ + 0.25SiC + 0.43C (11)

**Figure 6:**
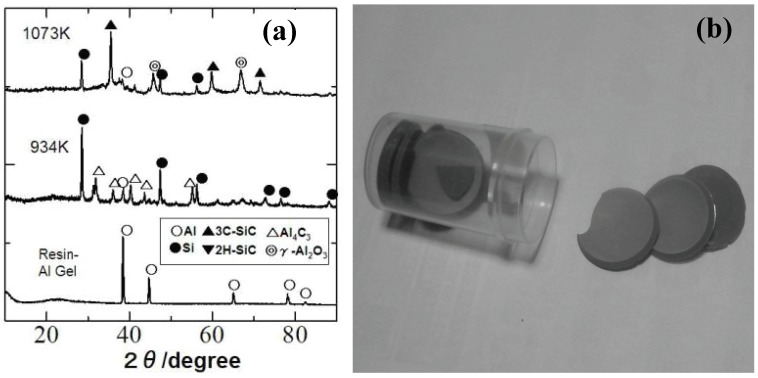
(a) Dried gel sheets derived from the silicone resin (YR3370^TM^) and 3 μm Al particles (Al/Si molar ratio of 1.0), (b) XRD patterns of composite gel sheet during pyrolysis.

In spite of the reduced carbon content in the starting silicone resin, the formed material contains a considerable amount of free carbon. In the pyrolysis of the resin - Al composite, the ceramizaton proceeds at quite low temperature, as shown in the corresponding XRD patterns [Figure 6(a) and (b)]. Al_2_O_3_, SiC and free Si formation at 800 °C can be explained by the following equation:

SiO_1.5_C_0.68_ + Al → 0.68SiC + 0.5Al_2_O_3_ + 0.32Si (12)

In this case, the thermodynamically stable phases must contain free silicon instead of free carbon. We remember the classic thermite process from the observed phase combination, but the absolute temperature does not show sharp rise during the heat treatment. Perhaps, the maintained organic nature of the surrounding silicone resin matrix plays an important role for promoting the low temperature ceramization. It is also curious that free silicon and Al_4_C_3_ are observed at 661 °C with disappearance of aluminum metal, while Al_2_O_3_ and SiC are absent in XRD patterns. Amorphous Al oxide formation, metallic liquid phase formation with eutectic Si-Al composition or disproportionation reaction in the Si-O-(C) matrix is considerable mechanism for explaining the free Si formation. Identification of a clear mechanism, however, requires further study.

The unique properties of the nano scale interface between pyrolyzed silicone resins and nano size powders are now becoming apparent. Riedel *et al*. demonstrated mullite phase formation at 1,300–1,500 °C in the case of a Wacker-Belsil PMS MK^TM^ and γ-alumina combination. This temperature region is far lower than temperature of liquid phase formation in SiO_2_-Al_2_O_3_ system. By chemical modification of the nano alumina surface, mixing, warm pressing and pyrolysis in an inert atmosphere, nano composites of alumina (60–160 nm) and silicon carbide (1–8 nm) were successfully obtained. Ionescu *et al*. reported that incorporation of a large amount of zirconia nano powder in Si-O-C matrix remarkably suppressed degradation process of the synthesized composites at 1,600 °C, which is caused by carbothermal reduction and crystallization processes [23]. A detailed mechanism is not defined, but some chemical interaction is suggested for Si atoms in Si-O-C and Zr atoms in zirconia. Even in such “passive oxide filler” cases, it is certain that interface can become “active” under some chemical or physical conditions.

Since commercialized silicone resins with high ceramic yield usually conserve some amount of silanol groups in their molecular structures, the resins often accept chemical modification by various alkoxides. The uses of the alkoxides as cross-linking agents guarantee the atomic level distribution of introduced elements in the starting resin networks, and homogeneous structure evolution during the pyrolysis is expected. In particular, high temperature properties of pyrolyzed materials, like oxidation resistance, corrosion resistance or creep resistance, are probably influenced by additional elements. In the case of the Al alkoxide addition in the PMS MK^TM^, the alkoxide acts as a cross-linking agent and suppresses the crack formation process during the pyrolysis [26]. In the high temperature region, 1,200–1,600 °C, the role of SiO_2_-Al_2_O_3_ liquid phase formation for reduced cracking is suggested. Zr(O^n^Pr)_4_ is also effective as a cross-linking agent, and nano domains of zirconia were successfully dispersed in the Si-O-C matrix after pyrolysis [23]. High temperature stability at 1,600 °C was also improved. A high Zr amount was, however, required even in this case.

## 8. Summary

In this review, I have tried to classify the various silicone resins. The most important feature is the main chain structure of the original silicones, because the ceramic yield of the silicone resin depends on the rate of cyclic oligomer expulsion during the heating. The second point is the resulting chemical composition of the pyrolysis products. The balance of the formed phases (SiC, SiO-CO, free carbon and SiO_2_) and the microstructure after the simple pyrolysis are determined by the chemical composition of SiC_x_O_y_ amorphous. The third point is the viscoelastic natures of the prepared silicones. Usually, the silicone resins are viscous liquids and often accept cross-links. The obtained resins or cross-linked elastomer can accept relatively high extension without cracks as compared with ordinary ceramic precursors, like polycarbosilane, polysilazane and polysilanes. This character is useful to shape the starting materials into desired forms by molding, casting, injection, impregnation or extrusion. Shape stability during pyrolysis is also promising, if the chain migration is permitted at least locally even during the pyrolysis.

The high carbon or high oxygen contents in silicone resins have been thought to decrease the heat resistance of the resulting SiO_x_C_y_ amorphous. Even in an inert atmosphere, degradation of the amorphous proceeds with CO-SiO evolution beyond 1,400 °C. Recently, however, the adverse effect of such excess carbon and oxygen can be reduced principally by using the special fillers or cross-linking agents as described.

In spite of their undoubted usefulness, silicone resins have been described as supporting players in ceramic synthesis processes. I wish that this review will provide readers with some knowledge of the various silicone resin natures from the viewpoints of the polymer science and inorganic chemical reaction. Silicone resins are old and classic materials developed at the early years of the 20th century. The materials, however, hold hidden potential even at present, and are possibly key materials in the 21st century for promoting the precursor method availability in wide industrial applications.
